# A Case of Misdiagnosed Pediatric Congenital Nephrogenic Diabetes Insipidus due to AQP2 Variants

**DOI:** 10.1155/crpe/9068249

**Published:** 2026-10-08

**Authors:** Valeria Chirico, Fabiola Cassaro, Serena Sbilordo, Antonio Lacquaniti, Lorena Silipigni, Lucia Marseglia, Karol Galletta, Francesca Granata, Paola Tirelli, Grazia Cirillo, Salvatore Arena, Pierluigi Marzuillo, Roberto Chimenz

**Affiliations:** ^1^ Department of Human Pathology of Adult and Childhood “Gaetano Barresi” University of Messina Messina Italy unime.it; ^2^ Department of Biomedical and Dental Sciences and Morpho-Functional Imaging University of Messina Messina 98124 Italy unime.it; ^3^ Department of Internal Medicine, Nephrology and Dialysis Unit Papardo Hospital Messina Italy aopapardo.it; ^4^ Department of Woman, Child and of General and Specialized Surgery University of the Study of Campania “Luigi Vanvitelli” Via Luigi De Crecchio 2 Naples Italy

**Keywords:** AQP2, children, congenital nephrogenic diabetes insipidus, polyuria

## Abstract

**Background:**

Congenital nephrogenic diabetes insipidus (CNDI) is a rare disorder of renal water handling, often presenting in early infancy with severe polyuria and hypernatremia. Differentiating CNDI from central diabetes insipidus (CDI) remains challenging, and delayed identification may result in inappropriate therapy, leading to adverse growth and neurodevelopmental outcomes.

**Case Presentation:**

We present the case of an 8‐month‐old male infant with a history of suspected CDI since birth. He was treated with desmopressin (dDAVP) without clinical benefit. The absence of the posterior pituitary bright spot at magnetic resonance imaging supported the CDI diagnosis. Considering persistent polyuria with recurrent episodes of hypernatremia and low urine osmolality, a diagnosis of CNDI was proposed, and plasma copeptin (CP) levels were determined. Measurement of CP showed markedly increased concentrations, suggesting renal tubular resistance to vasopressin. dDAVP therapy was discontinued and patient’s management was adjusted to include hydrochlorothiazide therapy and a high‐calorie diet restricted in sodium and protein. The patient showed progressive clinical improvement with reduction of polyuria and stabilization of serum sodium levels, although linear growth delay persisted. A final diagnosis of CNDI was confirmed by molecular analysis that identified rare compound heterozygous variants in the AQP2 gene, c.85G > A variant (maternal) and c.553C > G (paternal).

**Conclusions:**

This case highlights the diagnostic pitfalls of CNDI in infancy and underscores the pivotal role of early CP assessment and molecular genetic testing in distinguishing CNDI from CDI. Prompt recognition of CNDI is essential to avoid ineffective dDAVP therapy and to initiate a targeted multidisciplinary approach. Rare *AQP2* mutations further expand the genotypic spectrum of CNDI and emphasize the need for heightened clinical awareness and early referral to specialized centers.

## 1. Background

Congenital nephrogenic diabetes insipidus (CNDI) is a rare renal tubular disorder with an estimated prevalence of 1 in 25.000 individuals [1]. CNDI typically presents in early infancy, with nearly 60% of patients diagnosed within the first year of life, while only 6% are identified in adulthood [2].

It is characterized by the kidneys’ inability to concentrate urine in response to vasopressin (AVP), leading to severe polyuria, compensatory polydipsia, and risk of dehydration [3].

In 2022, a consensus working group proposed the term “Arginine Vasopressin Resistance (AVP‐R)” to better reflect the underlying pathophysiology more accurately [4].

Clinical manifestations of CNDI include recurrent fever, vomiting, and irritability within 1 week from birth. Failure to thrive and developmental delay are often present due to recurrent hypernatremia and dehydration [5].

Laboratory results characteristic of CNDI include urine output > 4 mL/kg/h, serum sodium > 170 mmol/L, serum osmolality > 300 mOsm/kg, urine osmolality < 300 mOsm/kg, and a urine specific gravity < 1,005 g/mL [6].

If not recognized promptly, these episodes may lead to acute kidney injury and, in the long term, to urological complications related to chronic polyuria, such as megacystis, hydronephrosis, and hydroureter [6–8].

Neurodevelopmental and behavioral disorders, including attention‐deficit hyperactivity disorder and mental health problems, have also been reported [9].

The diagnosis and management of CNDI is very challenging and requires an integrated, multidisciplinary approach.

We report the case of an 8‐month‐old infant with failure to thrive, recurrent vomiting, persistent pyrexia, and hypernatremia in order to highlight the diagnostic challenges of this condition during the first months of life and to provide general pediatricians with practical guidance for its management.

## 2. Case Presentation

An eight‐month‐old male child was admitted to our Pediatric Nephrology Unit for evaluation of failure to thrive, recurrent vomiting, and persistent pyrexia.

He was born at a referring center at 41 weeks of gestation via an emergency cesarean delivery due to abnormal fetal heart rate patterns, weighing 4270 g with a length of 52.5 cm.

There were no signs of intrauterine distress or postnatal asphyxia. Family history was negative for polyuria, polydipsia, or inherited disorders.

On the fourth day of life, he presented with fever, diarrhea, and clinical signs of dehydration. Laboratory tests revealed hypernatremia (Na 171 mmol/L), elevated serum osmolality (369 mOsm/L), and low urine specific gravity (PS 1003).

A presumptive diagnosis of central diabetes insipidus (CDI) was made by the local clinical team, and intranasal dDAVP was started (10 mcg/day).

The intranasal route was chosen due to the neonatal age and the unavailability of alternative formulations suitable for oral or sublingual administration at that time.

Despite partial reduction in urine output and clinical stabilization under initial therapy at the referring center, severe hypernatremia persisted (serum sodium: 160 mmol/L), initially attributed to suboptimal intranasal absorption during the neonatal period.

Imaging studies showed normal renal ultrasound findings and structurally normal brain parenchyma and neurohypophysis on magnetic resonance imaging (MRI). Genetic testing was also performed.

Over the following months the patient exhibited persistent anorexia, vomiting, and failure to thrive, leading his parents to refer him to our tertiary care center.

### 2.1. Clinical Examination and Investigations

Upon admission to our center at 8 months of age, the infant presented with a body temperature of 38°C, dry skin, and clinical signs of dehydration. Anthropometric evaluation revealed a weight of 6000 g (< 3rd) and a length of 65 cm (3rd) according to World Health Organization growth charts.

The neurologic examination showed a delay in postural stages.

Despite receiving intranasal dDAVP at a daily dose of 3.5 mcg/kg/day, no clinical or biochemical improvement was observed.

A routine blood test showed sodium levels of 155 mmol/L, plasma osmolality of 309 mOsm/L, urine specific gravity of 1004, and urinary osmolality of 251 mOsm/kg; his urine output was 4 L/day.

Elevated serum renin (1354 pg/mL) and aldosterone (1253 pg/mL) levels was observed.

Clinical, laboratory investigations are listed in Table 1.

**TABLE 1:** Clinical and laboratory characteristics of patient at birth, at admission to our centre and follow‐up.

	Patient
At birth	
Sex	Male
Gestational age, week	41
Weight, gr	4270
Height, cm	52.5
Symptoms	Diarrhea and dehydration 1 week after birth
Serum Na, mmol/L (range 134–145)	171
Aldosterone, pg/mL (range 49–175)	1100
Renin, pg/mL (range 2.71–16.51)	580
Urine specific gravity	1003
Urologic complications	none
Brain MRI	none
Treatment	dDAVP intranasal (10 mcg/day)
At admission	
Age, months	8
Height, cm (P)	65 (< 3^rd^)
Weight, gr (P)	6000(< 3^rd^)
Symptoms	Poor body weight gain, vomit, and fever
Serum Na, mmol/L (range 134–145)	159
Serum co‐peptin, pmol/L	438.1
Aldosterone, pg/mL (range 49–175)	1253
Renin, pg/mL (2.71–16.51)	1354
FSH, m(IU)mL(< 22.0)	2.61
LH, m(IU)mL (< 11.90)	< 3.0
Plasma osmolality, mOsm/L	309
Urine osmolality, mOsm/kg	251
Diuresis	4 L/day
Urinary specific gravity	1004
Urologic complications	None
Brain MRI	Absence of physiological hyperintensity in T1
Variant	*AQP2* variant(c.85G > A; c.553C > G)
Treatment	Stopped DA and started oral HZ
Psychomotor therapy	
Diet (low‐protein, low sodium diet)	
Solute‐free fluid intake	
Follow‐up	2 years
Height cm (P)	9.600 (< 3^rd^)
Weight gr (P)	82 cm (< 3^rd^)
Diuresis	2 L/day

Abdominal ultrasonography showed minimal and nonspecific dilatation of the right renal pelvis.

Brain MRI revealed an anteriorized and stretched pituitary stalk with the absence of the typical posterior pituitary hyperintensity on T1‐weighted images (“bright spot”) (Figure 1).

**FIGURE 1:**
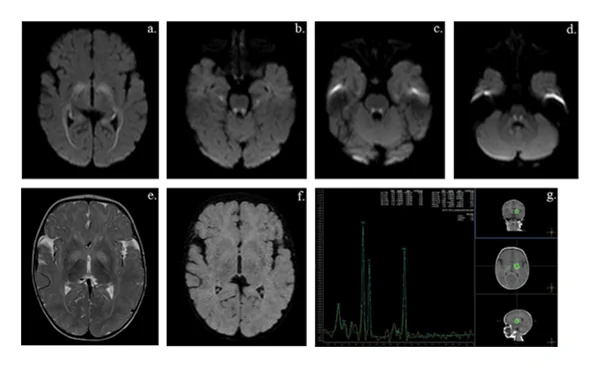
Magnetic resonance imaging (MRI) at clinical onset. (a) Axial diffusion‐weighted imaging (DWI) at the level of the basal ganglia showing symmetric areas of restricted diffusion in the globi pallidi (red arrows). (b) Axial DWI at a slightly lower level demonstrating persistent bilateral diffusion restriction involving the globi pallidi (red arrows). (c) Axial DWI at the level of the midbrain/upper pons showing symmetric hyperintense lesions along the medial lemniscus pathways (red arrows). (d) Axial DWI at the level of the dorsal pons demonstrating bilateral restricted diffusion within the pontine region (red arrows). (e) Axial T2‐weighted fast spin echo (FSE) image showing bilateral symmetric hyperintensities in the globi pallidi (arrowheads), consistent with demyelination. (f) Axial fluid‐attenuated inversion recovery (FLAIR) image confirming corresponding bilateral hyperintensities in the globi pallidi (arrowheads). (g) MRS (intermediate TE = 144 ms) shows markedly elevated Cho/Cr ratio and decreased NAA/Cr ratio in the area of active dysmyelination: Cho/Cr = 1.7.

Screening of the anterior pituitary axes demonstrated a basal Follicle‐Stimulating Hormone (FSH) level of 2.61 mIU/mL (reference range: < 22.0 mIU/mL) and Luteinizing hormone (LH) level of < 3.0 mIU/mL (reference range: < 11.90 mIU/mL).

### 2.2. Management and Follow‐Up

Given the persistent polyuria and hypernatremia (Na 160 mmol/L) despite dDAVP treatment, CNDI was suspected.

A formal water deprivation test (WDT) was not performed due to the patient’s young age and clinical instability. Furthermore, the presence of significant hypernatremia represented an absolute contraindication to the procedure. Given the persistent nonresponsiveness to dDAVP, plasma copeptin (CP) was assessed. Levels were profoundly elevated at 438.1 pmol/L (normal: < 10.0 pmol/L), confirming the diagnosis of CNDI. Intranasal dDAVP treatment was discontinued, and oral hydrochlorothiazide (HZ) was started at 1 mg/kg, progressively increased to 3 mg/kg.

Nutritional management consisted of a low‐protein and low‐sodium diet with unrestricted access to solute‐free water. Although nasogastric tube feeding was recommended to address the persistent growth failure, the parents declined the intervention.

A neurological examination confirmed the developmental delay in motor milestones, and psychomotor therapy was promptly initiated.

Following therapeutic adjustments, significant clinical and laboratory improvements were observed, including the normalization of body temperature, hydration status, serum sodium levels, serum osmolality, urine osmolality, and urine specific gravity. The parents were instructed to supplement water between milk and food consumption.

Electrolyte levels in the urine and serum were monitored monthly and followed up regularly.

At 14 months of age, follow‐up brain MRI demonstrated improved visualization of the neurohypophysis region with partial restoration of the posterior pituitary signal (Figure 2).

**FIGURE 2:**
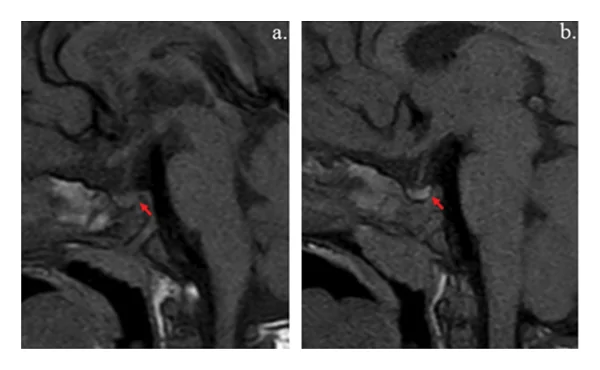
Pituitary gland MRI evaluation by Sagittal T1‐weighted Fast Spin Echo (FSE) images at diagnosis (a) and after medical treatment (b). (a) Lack of the posterior pituitary bright signal (arrow). (b) Partial recovery of the characteristic bright signal of the posterior lobe of the pituitary gland (arrow).

No abnormalities were observed at the renal color Doppler ultrasound.

The patient showed a good therapeutic response, with diuresis of 2 L/day and improved laboratory findings. At the time of the last follow‐up, the patient was 2 years of age, weighed 9.600 kg (2^nd^ percentile), and was 82 cm (2^nd^ percentile). No urological complications were observed, and global developmental milestones progressively improved (Figure 3).

**FIGURE 3:**
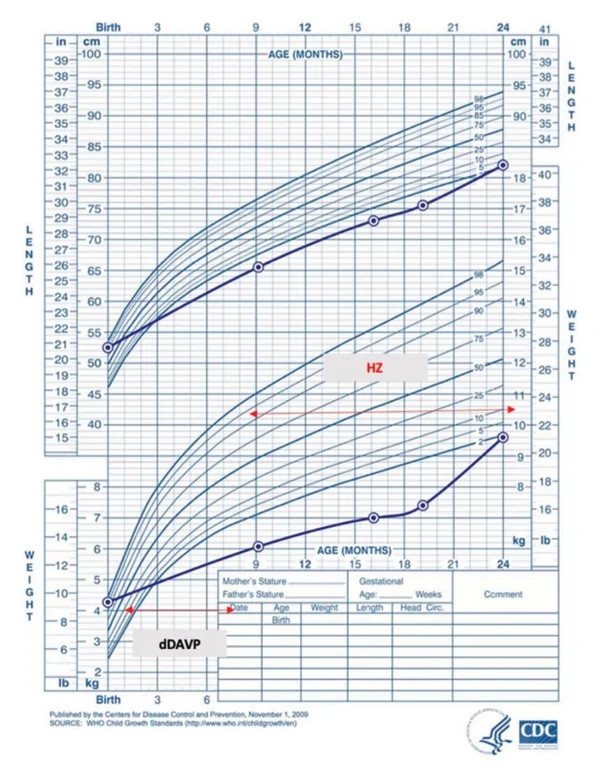
Growth chart of the patient. The slow progress of height and weight and the course of therapy in our patient are shown. At the age of 9 months, after controlling his disease by stopping dDAVP and starting HZ therapy, the patient showed catch‐up. Abbreviations: HD = hydrochlorothiazide; dDAVP = desmopressin.

### 2.3. Genetic Analysis

Genetic testing revealed compound heterozygous variants in the AQP2 gene, c.85G > A variant (maternal) and c.553C > G (paternal).

### 2.4. In Silico Analysis

Both variants have already been previously described in patients with CNDI [10, 11].

According to the American College of Medical Genetics and Genomics (ACMG) guidelines [12, 13], the two variants in the AQP2 gene (NM_000486), c.85G > A p.Gly29Ser and c.553C > G p.Pro185Ala, both extremely rare (MAF 0.1%), were classified as likely pathogenic based on the ACMG criteria PM2 (absent or present at an extremely low frequency in population databases), PM3 (the variants were demonstrated to be in trans by parental segregation analysis), and PP3 (multiple computational prediction tools supported a deleterious effect).

Moreover, we assessed these variants using three in silico prediction tools: Combined Annotation Dependent Depletion (CADD), which integrates multiple annotations to estimate the deleteriousness of genetic variants; Sorting Intolerant From Tolerant (SIFT), which predicts whether an amino acid substitution affects protein function based on sequence homology and physicochemical properties; and Rare Exome Variant Ensemble Learner (REVEL), an ensemble method that combines multiple prediction algorithms to estimate the pathogenicity of rare missense variants. The variants c.85G > A and c.553C > G showed scores of 26.8 and 24.9 for CADD, 0.02 and 0.0 for SIFT, and 0.861 and 0.957 for REVEL, respectively. This supports a deleterious and likely pathogenic effect.

## 3. Discussion

CNDI is a rare genetic disorder characterized by renal tubular insensitivity to AVP, leading to an inability to concentrate urine despite normal or elevated AVP levels.

The diagnosis of CNDI in neonates and young infants is inherently difficult because symptoms are often nonspecific, and clinical conditions are frequently unstable.

Early infancy presentation, marked by vomiting, irritability, recurrent fever, and growth retardation, often leads to diagnostic delay, as symptoms overlap with more common pediatric conditions. Therefore, it is important for general pediatricians to keep in mind that, when an infant presents with these clinical manifestations, urinary output should be carefully measured to detect polyuria and orient the diagnosis toward tubulopathies. In addition, if hypernatremia is present and blood pH and bicarbonate levels are normal, the diagnostic suspicion should shift toward DI.

The baseline laboratory findings of our patient on the fourth day of life, characterized by severe hypernatremia (Na 171 mmol/L), elevated serum osmolality (369 mOsm/L), and inappropriately low urine specific gravity (1.003) established a diagnosis of DI.

Nevertheless, distinguishing between CDI and CNDI during the immediate neonatal period remains an intricate clinical dilemma. Given its significantly higher prevalence relative to the extreme rarity of CNDI, CDI is typically the initial diagnostic consideration.

Consequently, clinicians often misattribute neonatal DI to transient postnatal factors, subclinical perinatal hypoxic insults, or a delayed functional maturation of the neurohypophysis.

The classic diagnostic approach relies on the WDT to differentiate DIfrom primary polydipsia, followed by administration of dDAVP to distinguish CNDI from CDI [14].

In this context, however, the WDT is rarely feasible or safe, and repeated venous sampling may be technically challenging. Consequently, in clinical practice diagnostic evaluation could be simplified, and dDAVP may be empirically initiated without adequate biochemical confirmation, increasing the risk of misclassification.

In our case, this diagnostic pitfall was further compounded by different factors.

Initial clinical management and the interpretation of the therapeutic response occurred prior to the patient’s referral to our unit.

The patient’s endocrine profile at birth presented a significant interpretive challenge regarding the activation of the renin–angiotensin–aldosterone system (RAAS).

From a pathophysiological standpoint, markedly elevated renin and aldosterone levels at birth, compounded by chronic dehydration, served as a pivotal diagnostic clue rather than a confounding factor. Rather than signaling a primary adrenal endocrinopathy, these biochemical findings reflected a profound compensatory activation of the RAAS secondary to chronic volume depletion, pointing directly toward renal resistance to AVP.

Following the intranasal dDAVP therapy, the referring clinical team observed a partial reduction in serum sodium levels (from 171 to 160 mmol/L), accompanied by an apparent decrease in urine output. This was misinterpreted as a partial therapeutic response, leading to the continuation of the presumptive CDI diagnosis. In neonates, the intranasal route exhibits highly erratic, unpredictable, and suboptimally low mucosal absorption due to anatomical constraints and variable mucosal hydration.

This clinical course underscores a vital message: persistent hypernatremia despite dDAVP therapy constitutes an immediate red flag, prompting clinicians to reconsider CDI diagnosis and immediately investigate for CNDI.

An overreliance on neuroimaging also contributed to the initial diagnostic error in this patient. Brain MRI performed during the initial workup showed the absence of the typical posterior pituitary hyperintensity on T1‐weighted images (the “bright spot”), a structural finding traditionally associated with CDI. However, it is crucial to recognize that the absence of the “bright spot” is not pathognomonic for CDI; up to 39% of patients with primary polydipsia and a significant proportion of patients with CNDI or even healthy infants may lack this hyperintense signal [14–16].

In chronic polyuric states like CNDI, the constant, maximal stimulation of the hypothalamic‐pituitary axis leads to unremitting exocytosis of neurosecretory granules, depleting the vasopressin stores that generate the T1 signal.

A partial restoration of this hyperintense signal observed in our patient’s follow‐up MRI at 14 months confirms that once appropriate hydration and fluid‐electrolyte balance were achieved through targeted CNDI therapy, the unremitting demand on the neurohypophysis subsided, allowing for the replenishment of neurosecretory granules.

Therefore, pituitary imaging should be interpreted with caution and not be utilized as a definitive exclusion criterion for nephrogenic forms.

Given the prohibitive risk of performing a WDT in neonates, and the potential for misleading neuroimaging findings, a clear diagnostic impasse often arises in clinical practice.

To resolve this diagnostic challenge, plasma CP has emerged as a revolutionary and accurate diagnostic biomarker.

CP is co‐synthesized with AVP from the pre–pro‐AVP precursor in response to increasing osmolality and is released into the circulation in equimolar concentrations. Unlike AVP, which is highly unstable and rapidly cleared, CP is stable in plasma and easily measured [8].

In adult cohorts, prior studies demonstrated that a basal CP level greater than 21.4 pmol/L without prior fluid restriction strongly supports a diagnosis of CNDI, effectively rendering the WDT unnecessary [9, 17].

In the pediatric population, published validation data strongly support its utility, demonstrating that baseline and stimulated CP measurements provide excellent sensitivity and specificity for identifying renal vasopressin resistance and diagnosing polyuria‐polydipsia syndromes in children [17].

In our patient, the plasma CP level was measured upon admission in the setting of severe hypernatremia (Na 160 mmol/L) and marked hyperosmolality. Because severe hypernatremia itself acts as a potent endogenous osmotic stimulus, the baseline value (438.1 pmol/L) represented an effectively “stimulated” measurement rather than a random, uninterpretable basal level. The massive elevation of this biomarker provided unequivocal biochemical proof of extreme renal AVP resistance, definitively establishing the diagnosis of CNDI.

Identification of this biomarker enabled an immediate therapeutic pivot, prompting the discontinuation of ineffective intranasal dDAVP in favor of oral HZ therapy.

While CP confirmed the diagnosis of renal AVP resistance, identifying the exact genetic cause required formal molecular testing.

From a genetic standpoint, approximately 90% of CNDI cases are caused by X‐linked variants in the *AVPR2* gene. Therefore, in any male infant presenting with hypernatremia, polyuria, and failure to thrive, CNDI must be suspected immediately. Despite our patient’s male gender and classic presentation, the referring center initially overlooked this statistical likelihood, opting for empirical dDAVP trials under the assumption of a more common central etiology.

This case highlights a critical diagnostic pitfall: overreliance on empirical treatment without definitive confirmation leads to severe therapeutic delays. Primary care clinicians must recognize that given the overwhelming prevalence of X‐linked forms, a male infant with unremitting DI features should be treated as having a nephrogenic origin until proven otherwise.

The remaining 10% of CNDI cases are due to autosomal recessive or dominant variants in the *AQP2* gene [4]. Clinical presentation does not significantly differ between these genotypes, making molecular testing essential for definitive diagnosis and genetic counseling. In about 2% of patients, no identifiable genetic cause is found [14]. The *AQP2* variants are known to cause misfolding and endoplasmic reticulum retention, preventing aquaporin insertion into the apical membrane of collecting duct cells and defining autosomal recessive CNDI as a Class II trafficking disorder [10, 11], highlighting the importance of correct protein folding and post‐translational modification in aquaporin‐mediated water transport [11].

To date, approximately 70 disease‐causing variants have been reported in AQP2, with missense mutations accounting for 77% [15].

In our case, molecular diagnosis revealed compound heterozygous *AQP2* variants (c.85G > A; p.Gly29Ser and c.553C > G; p.Pro185Ala), both extremely rare (MAF 0.1%).

As illustrated by this complex clinical course, the long‐term management of CNDI requires an integrated multidisciplinary team, including a pediatric nephrologist, geneticist, nutritionist, and endocrinologist, to avoid secondary complications and optimized outcomes.

The management of our patient was adjusted to combine oral HZ therapy, breastfeeding on demand, a high‐calorie diet, and strict low‐sodium and low‐protein dietary restrictions to minimize the renal solute load, due to the severity of the initial growth failure.

A nutritional support with gastrostomy tube placement was indicated, though the family declined. In a large North American cohort of 66 children with CNDI, failure to thrive was the most frequent indication for gastrostomy tube placement, and although catch‐up growth was achievable, many patients continued to show mild growth deficits at follow‐up [18].

Similarly, Sharma et al. reported that while most CNDI patients eventually reach a near normal final adult height, their mean height at follow‐up remains slightly below the population average (−0.9 SDS) [19].

Nevertheless, under rigorous multidisciplinary care, our patient achieved an overall highly favorable prognosis, demonstrating progressive catch‐up growth, significantly reduced polyuria, normal developmental milestone progression, and a total absence of secondary urological complications.

These observations are consistent with previous literature confirming that a timely diagnosis, appropriate hydration, adequate support, and individualized medical therapy are the key determinants of optimal long‐term outcomes [9].

## 4. Conclusions

In conclusion, our case report highlights several key messages that may guide general pediatricians in the management of infants presenting with hypernatremia and polyuria: CNDI should be considered in the differential diagnosis of growth failure and feeding difficulties in infancy, as chronic thirst may reduce caloric intake. Therefore, urine output should always be carefully measured in these patients; in a male infant presenting with hypernatremia, polyuria, and failure to thrive, CNDI should be strongly suspected, as the condition is most commonly X‐linked; the persistence of hypernatremia despite dDAVP administration should not be ignored, prompting immediate etiological reconsideration; CP levels may help support the clinical suspicion of CNDI while awaiting genetic confirmation; the absence of the typical posterior pituitary hyperintensity on T1‐weighted MRI is not sufficiently sensitive to support a diagnosis of CDI in infants.

Keeping these clues in mind is crucial to achieving a timely and accurate diagnosis, which can prevent long‐term complications, optimize growth, promote neurodevelopment, and improve quality of life.

**Nomenclature**
ACMG: American College of Medical Genetics and Genomics
ADH: Antidiuretic hormone
AQP2: Aquaporin‐2
AVP: Arginine vasopressin
AVP‐R: Arginine vasopressin resistance
CADD: Combined annotation dependent depletion
CDI: Central diabetes insipidus
CNDI: Congenital nephrogenic diabetes insipidus
CP: Copeptin
DA (dDAVP): Desmopressin
DI: Diabetes insipidus
FSE: Fast spin echo
HZ: Hydrochlorothiazide
LH: Luteinizing hormone
MAF: Minor allele frequency
MRI: Magnetic resonance imaging
MRS: Magnetic resonance spectroscopy
NSAIDs: Nonsteroidal anti‐inflammatory drugs
PP: Primary polydipsia
REVEL: Rare exome variant ensemble learner
SDS: Standard deviation score
SIFT: Sorting intolerant from tolerant
TD: Thiazide diuretics
TZ: Thiazide
WDT: Water deprivation test
WHO: World Health Organization

## Author Contributions

Conceptualization: Valeria Chirico and Roberto Chimenz; methodology and validation: Valeria Chirico, Roberto Chimenz, and Fabiola Cassaro, data curation: Serena Sbilordo, Lorena Silipigni, Lucia Marseglia, Karol Galletta, Salvatore Arena, and Francesca Granata, writing–original draft preparation: Valeria Chirico, Fabiola Cassaro, Paola Tirelli, and Antonio Lacquaniti; writing–review and editing, Valeria Chirico, Fabiola Cassaro, Grazia Cirillo, and Pierluigi Marzuillo; supervision: Valeria Chirico and Pierluigi Marzuillo.

## Funding

The authors received no funding.

Open access publishing facilitated by Universita degli Studi di Messina, as part of the Wiley ‐ CRUI‐CARE agreement.

## Disclosure

All authors have read and agreed to the published version of the manuscript.

## Ethics Statement

Ethics approval was not required for this case report according to institutional guidelines.

## Consent

Written informed consent for participation and publication was obtained from the patient’s parents.

Informed consent was obtained from the patient’s parents.

## Conflicts of Interest

The authors declare no conflicts of interest.

## Supporting Information

Additional supporting information can be found online in the Supporting Information section.

## Supporting information


**Supporting Information** CARE‐checklist‐English‐2013.

## Data Availability

The data that support the findings of this study are available on request from the corresponding author. The data are not publicly available due to privacy or ethical restrictions.
