# Supplementary material for: A Case of Misdiagnosed Pediatric Congenital Nephrogenic Diabetes Insipidus due to AQP2 Variants
Source: Case Rep Pediatr. 2026 Oct 8;2026:9068249. doi: 10.1155/crpe/9068249 (PMC13647111; doi:10.1155/crpe/9068249)
Supplement: Supplementary file 1 [file CRPE-2026-9068249-s001.pdf]

CARE case report guidelines

CARE Checklist of information to include when writing a case report

(cc) BY-NC-ND

Topic	Item	Checklist item description	Reported on Line
Title	1	The diagnosis or intervention of primary focus followed by the words "case report"	1
Key Words	2	2 to 5 key words that identify diagnoses or interventions in this case report, including "case report"	54
Abstract (no references)	3a	Introduction: What is unique about this case and what does it add to the scientific literature?	30-34
	3b	Main symptoms and/or important clinical findings	35-47
	3c	The main diagnoses, therapeutic interventions, and outcomes	46-47
	3d	Conclusion—What is the main "take-away" lesson(s) from this case?	48-52
Introduction	4	One or two paragraphs summarizing why this case is unique (**may include references**)	55-77
Patient Information	5a	De-identified patient specific information.	82-86
	5b	Primary concerns and symptoms of the patient.	87-88
	5c	Medical, family, and psycho-social history including relevant genetic information	142-144
	5d	Relevant past interventions with outcomes	87-88
Clinical Findings	6	Describe significant physical examination (PE) and important clinical findings.	101-113
Timeline	7	Historical and current information from this episode of care organized as a timeline	91-99
Diagnostic Assessment	8a	Diagnostic testing (such as PE, laboratory testing, imaging, surveys).	101-113 142-144
	8b	Diagnostic challenges (such as access to testing, financial, or cultural)	101-113
	8c	Diagnosis (including other diagnoses considered)	101-113
	8d	Prognosis (such as staging in oncology) where applicable	-
Therapeutic Intervention	9a	Types of therapeutic intervention (such as pharmacologic, surgical, preventive, self-care)	116-131
	9b	Administration of therapeutic intervention (such as dosage, strength, duration)	116-131
	9c	Changes in therapeutic intervention (with rationale)	129-131
Follow-up and Outcomes	10a	Clinician and patient-assessed outcomes (if available)	134-140
	10b	Important follow-up diagnostic and other test results	137-140
	10c	Intervention adherence and tolerability (How was this assessed?)	134-140
	10d	Adverse and unanticipated events	139-140
Discussion	11a	A scientific discussion of the strengths AND limitations associated with this case report	165-176
	11b	Discussion of the relevant medical literature **with references**.	165-232
	11c	The scientific rationale for any conclusions (including assessment of possible causes)	216-232
	11d	The primary "take-away" lessons of this case report (without references) in a one paragraph conclusion	235-245
Patient Perspective	12	The patient should share their perspective in one to two paragraphs on the treatment(s) they received	-
Informed Consent	13	Did the patient give informed consent? Please provide if requested	Yes ☒ No ☐
